# PVR—A Prognostic Biomarker Correlated with Immune Cell Infiltration in Hepatocellular Carcinoma

**DOI:** 10.3390/diagnostics12122953

**Published:** 2022-11-25

**Authors:** Wen-Feng Liu, Bing Quan, Miao Li, Feng Zhang, Ke-Shu Hu, Xin Yin

**Affiliations:** 1 Liver Cancer Institute, Zhongshan Hospital, Fudan University, 136 Yi Xue Yuan Road, Shanghai 200032, China; 2 National Clinical Research Center for Interventional Medicine, 136 Yi Xue Yuan Road, Shanghai 200032, China

**Keywords:** PVR, hepatocellular carcinoma, prognosis, immune cell infiltration

## Abstract

The poliovirus receptor (PVR) is a member of the immunoglobulin superfamily (Ig SF) and is essential for the promotion of cancer cell proliferation and invasion. However, the correlation between PVR expression and prognosis as well as immune infiltration in hepatocellular carcinoma (HCC) remains unclear. The expression level of PVR was quantified using the Tumor and Tumor Immunity Evaluation Resource (TIMER) and Sangerbox. The Gene Expression Omnibus (GEO) database was used to validate the PVR expression. The receiver operating characteristic (ROC) curve was used to evaluate the feasibility of using PVR as a differentiating factor according to the area under curve (AUC) score. A PVR binding protein network was built using the STRING tool. An enrichment analysis using the R package clusterProfiler was used to explore the potential function of PVR. Immune infiltration analysis was calculated with ESTIMATE algorithms. We also assessed the correlation between PVR expression and immune infiltration by the single-sample Gene Set Enrichment Analysis (ssGSEA) method from the R package GSVA and TIMER database. The results showed that PVR was commonly overexpressed in multiple types of tumors including HCC. The data of GSE64041 confirmed the same result. The ROC curve suggested that PVR could be a potential diagnostic biomarker. Additionally, high mRNA expression of PVR in HCC was significantly correlated with poor overall survival (OS) and relapse free survival (RFS). Results also indicated correlations between PVR mRNA expression with the level of infiltration immune cells including B cells, CD8+ T cells, cytotoxic cells, DCs, CD56dim NK cells, pDCs, and Th2 cells. Furthermore, the PVR level was significantly correlated with immune markers for immunosuppressive cells in HCC. In conclusion, PVR might be an important regulator of tumor immune cell infiltration and a valuable prognostic biomarker in HCC. However, additional work is needed to fully elucidate the underlying mechanisms.

## 1. Introduction

According to the Global Cancer Statistics 2020, liver cancer ranked sixth in incidence and fourth in mortality among all cancers [1,2]. In China, hepatocellular carcinoma (HCC) is the most common type of liver cancer. The incidence of HCC increases with age and is high at 40–60 years of age. There was a significant difference between the incidence of HCC in men and women, with a higher incidence in men. The etiology of HCC is complex, and its pathogenesis comprises the combination of biological, physical, chemical, and psychosocial factors. Hepatitis B virus (HBV), hepatitis C virus (HCV), aflatoxins, chemical drugs, ionizing radiation, genetic diseases, genetic mutations, and cirrhosis have been identified as major risk factors for HCC [3,4,5]. The onset of hepatocellular carcinoma is insidious and some early-stage HCC patients are often identified by imaging during unintentional physical examinations. Most patients have advanced-stage tumors when symptoms appear. Surgery, ablation, hepatic arterial chemoembolization, targeted drug, chemotherapy, immunotherapy, and radiation therapy are the current clinical treatment options for HCC. However, more than half of HCC patients are already in the middle and advanced stages at the time of diagnosis. Systemic treatment, especially immunotherapy, has become a crucial therapeutic option for these patients [6].

Immune checkpoint molecules play a role in co-stimulatory and co-inhibitory pathways. They tightly regulate the immune response and maintain self-tolerance under normal physiological conditions. It has been demonstrated that these pathways are dysregulated in tumors, resulting in an immunosuppressive microenvironment and immune evasion of cancer cells [7,8]. Programmed cell death 1 (PD-1)/PD-1 ligand 1 (PD-L1), cytotoxic T lymphocyte antigen 4 (CTLA-4), TIM3, and T-cell immunoreceptor with Ig and ITIM domains (TIGIT) are well-known immune-checkpoint molecules [9,10]. The poliovirus receptor (PVR), the ligand of TIGIT, has attracted much attention in recent years. PVR, a member of the immunoglobulin superfamily (IgSF), is a highly glycosylated type I transmembrane protein [11]. PVR can regulate signal transduction, cell adhesion, and proliferation by recruiting tyrosine phosphatase (SHP-2) [12,13,14]. In addition, PVR plays an important role in regulating the human immune system by binding to receptors such as CD226, CD96, and TIGIT [15]. An increasing number of studies have demonstrated a relationship between PVR and tumor progression in recent years. Multiple studies have shown that PVR was overexpressed in various cancers including lung adenocarcinoma, pancreatic cancer, ovarian cancer, myeloid leukemia, neuroblastoma, rectal cancer, and cholangiocarcinoma [16,17]. In tumors, the expression of PVR is also closely related to the degree of tumor differentiation and TNM stage [18]. Previous studies have also demonstrated that PVR can regulate the interaction between angiogenesis factor receptor 2 (VEGFR2) and integrin αvβ3, thus promoting the angiogenesis of tumor cells [19]. In addition, it is worth noting that PVR can serve as an important immunoregulatory protein and play a vital role in tumor cell immune surveillance and immune escape [20].

However, the function and mechanism of PVR in HCC progression remain poorly understood. Based on the expression of specific markers, we investigated the relationship between PVR expression and HCC prognosis as well as the level of immune cell in tumor stroma. The Tumor Immunity Estimation Resource (TIMER) database was used for more immune analysis. Our results revealed the crucial role of PVR in HCC prognosis. Moreover, PVR may regulate cancer immunity by regulating the level of immune cell infiltration.

## 2. Material and Methods

### 2.1. The Levels of PVR mRNA in Various Human Tumors

Comparisons of PVR expression in various tumor tissues and normal tissues were performed by the Sangerbox (http://vip.sangerbox.com/home.html (Last accessed on 15 November 2022)) and TIMER2.0 database (https://cistrome.shinyapps.io/timer (Last accessed on 15 November 2022)). GSE64041 (Platform: GPL6244) was downloaded from GEO datasets (https://www.ncbi.nlm.nih.gov/gds (Last accessed on 15 November 2022)) and used to obtain the HCC microarray data. To investigate the prognostic value of PVR in cancer patients, we used the R software version 3.6.3 (https://www.r-project.org/, The R Foundation (Last accessed on 15 November 2022)) package “ROC” for analysis and “ggplot2” for visualizations.

### 2.2. PVR Expression in Tumor Tissues Collected from HCC Patients

Tissues from 21 HCC patients who underwent surgical resection in Zhongshan Hospital were collected. Total RNA from tissues were extracted using the relevant purification kit (EZB, Suzhou, China). Reverse transcription and quantitative real-time PCR were conducted referring to the kit or the SYBR green mix (EZB, Suzhou, China) and the instructions of the PCR amplifier (Bio-Rad, CA, USA). The following primers were used for the genes indicated: β-actin, forward primer: GACTACCTCATGAAGATCCTCACC and reverse primer: TCTCCTTAATGTCACGCACGATT; PVR, forward primer: TGGAGGTGACGCATGTGTC and reverse primer: GTTTGGACTCCGAATAGCTGG. For immunohistochemistry, formalin-fixed paraffin-embedded sections (5 μm) were deparaffinized with xylene, rehydrated with a graduated series of ethanol, and rinsed with distilled water. After goat serum block, sections were stained with primary antibodies against PVR (ab267788, Abcam, Cambridge, UK) overnight, followed by incubation with the horseradish peroxidase-conjugated secondary antibody for 30 min at room temperature. Then, the sections were counterstained with hematoxylin.

### 2.3. Prognostic Analysis of PVR in HCC Patients

The Kaplan–Meier plot (http://kmplot.com/analysis/ (Last accessed on 15 November 2022)) was applied to evaluate the prognostic significance of PVR in HCC patients. Using the auto select best cutoff algorithm on the website, HCC patients were divided into two cohorts (high vs. low expression). Then, the hazard ratio (HR) and log-rank *p*-value were calculated.

### 2.4. PPI Network and GO/KEGG Analysis

A protein–protein interaction network (PPI) was constructed, and a module analysis was performed using STRING database (http://string-db.org/ (Last accessed on 15 November 2022)) with an interaction with a combined score of >0.4. To further clarify the function of PVR, GO and KEGG analysis using the top 100 genes most related with PVR were performed by the method described in a previous study [21].

### 2.5. Immune Landscape of PVR in HCC

Immune infiltration in HCC was assessed in different ways. First, we employed the Estimation of STromal and Immune cells in MAlignant Tumor tissues using the Expression data (ESTIMATE) algorithm (18), which scores each tumor sample for immune, stromal, and tumor purity. The R package “estimate“ was used to perform this analysis. Single-sample GSEA (ssGSEA) analysis was also performed to investigate the interactions between the tumor and immune cells. As for the correlation between PVR expression and the abundance of various immune cells, we calculated the *p*-values by performing the Wilcoxon rank-sum and Spearman’s rank. To consolidate the results of the analysis, we further used the correlation module in the Tumor Immune Estimation Resource (TIMER) database (http://timer.cistrome.org/ (Last accessed on 15 November 2022)) to analyze the association of PVR and markers of immune cells. Correlation coefficients were estimated using Spearman’s correlation. Gene expression levels were shown as log2 RSEM.

## 3. Results

### 3.1. PVR Was Upregulated in Pan-Cancer including HCC

**Figure 1:**
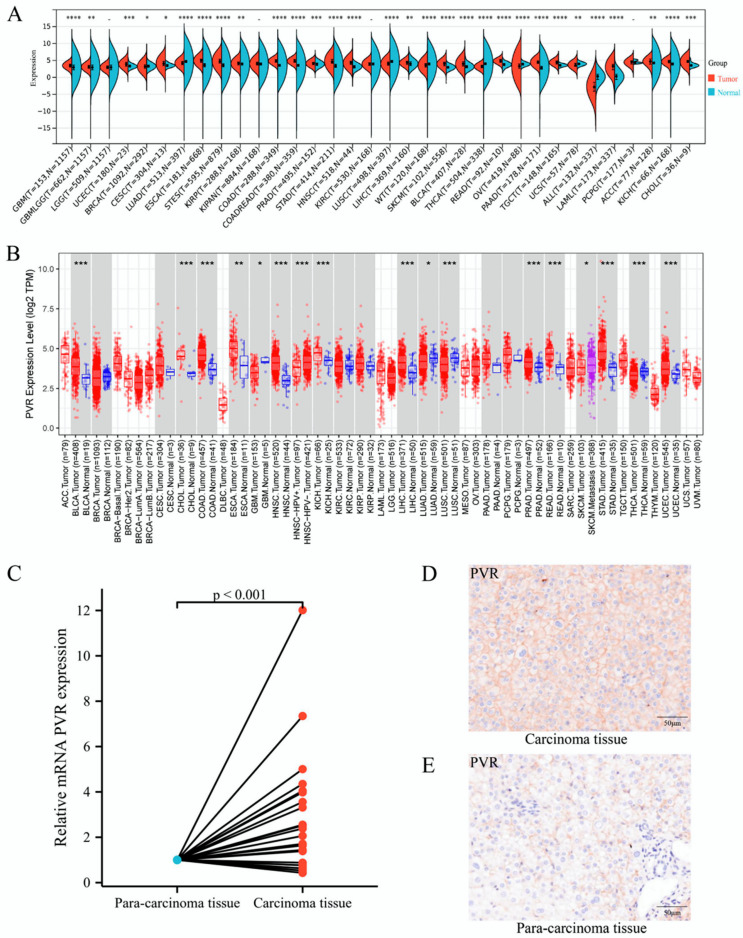
PVR was upregulated in pan-cancer including HCC. (**A**,**B**) The expression of PVR in pan-cancer obtained from the Sangerbox and TIMER database. (**C**) mRNA level of PVR in HCC and paracancer tissues collected from Zhongshan hospital. (**D**,**E**) The protein expression of PVR in the tumor tissue and paracancer tissues of HCC patients. * p value < 0.05; ** p value < 0.01; *** p value < 0.001; **** *p* value < 0.0001.

To determine the differences in PVR mRNA expression between normal and tumor tissues, the status of PVR mRNA was analyzed through Sangerbox and TIMER databases. The Sangerbox analysis demonstrated that the PVR mRNA was remarkably higher in glioblastoma multiforme, uterine corpus endometrial carcinoma, breast invasive carcinoma, cervical squamous cell carcinoma, endocervical adenocarcinoma, esophageal carcinoma, stomach and esophageal carcinoma, kidney renal papillary cell carcinoma, colon adenocarcinoma, prostate adenocarcinoma, stomach adenocarcinoma, head and neck squamous cell carcinoma, liver hepatocellular carcinoma, skin cutaneous melanoma, bladder urothelial carcinoma, rectum adenocarcinoma esophageal carcinoma, pancreatic adenocarcinoma, testicular germ cell tumors, acute myeloid leukemia, adrenocortical carcinoma, kidney chromophobe carcinoma, and cholangiocarcinoma in comparison to that in the corresponding normal tissue. However, PVR was significantly lower in Wilms’ tumor, thyroid carcinoma, ovarian serous cystadenocarcinoma, uterine carcinosarcoma, and acute lymphoblastic leukemia in comparison with that in the corresponding normal tissue (Figure 1A). The results from the TIMER database also demonstrated that the PVR expression was higher in most malignant tumors (Figure 1B). Total RNA was extracted from tumor tissues and the adjacent tissues of 21 patients with HCC undergoing surgery, and quantitative real-time PCR was performed. The results showed that the expression of PVR in HCC tumor tissues was higher than that in adjacent tissues (*p* < 0.001) (Figure 1C). Immunohistochemical staining data of tissues showed that the PVR protein expression was much higher in the tumor region than the paracancerous tissue (Figure 1D–E).

**Figure 2:**
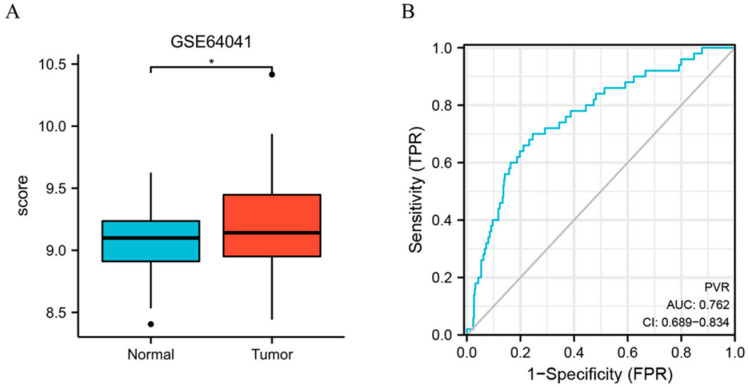
PVR expression in the GEO database. (**A**) PVR expression in the normal and tumor tissues in HCC from GSE64041. (**B**) ROC curve of PVR in HCC. * *p* < 0.05.

Furthermore, we assessed the PVR expression in HCC in GSE64041 (Platform: GPL6244), and the result confirmed that PVR was overexpressed in HCC tissue (Figure 2A). Additionally, we constructed the receiver operating characteristic (ROC) curve to evaluate the feasibility of using the PVR expression level to distinguish HCC from normal liver tissues. The area under the ROC curve (AUC) was 0.762, showing that the test was good.

### 3.2. Prognostic Significance of PVR Expression in HCC

**Figure 3:**
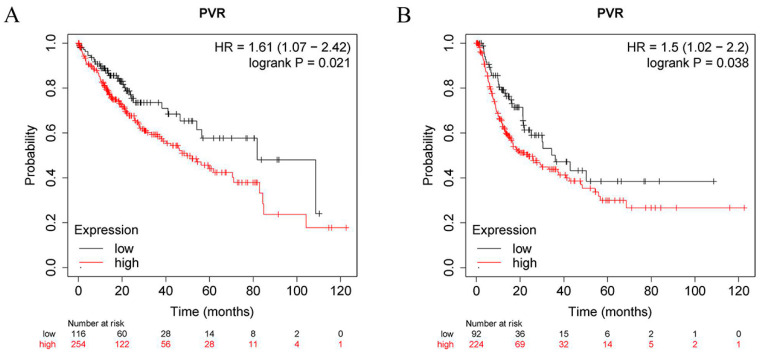
PVR has prognostic value in HCC. (**A**) OS between the high PVR group and low PVR group. (**B**) RFS between the high PVR group and low PVR group.

To determine the prognostic value of PVR, the correlation between the PVR mRNA levels and patient survival was investigated in HCC patients using the Kaplan–Meier plotter tool. The log-rank analysis revealed a significant correlation between the elevated PVR mRNA levels and poor OS and RFS in the HCC patients (*p* < 0.05) (Figure 3A,B). In addition, high PVR was also significantly associated with poorer OS in esophageal squamous cell carcinoma and lung adenocarcinoma (Supplementary Figure S1).

### 3.3. Association of PVR Expressions with Clinical Characteristics of HCC Patients

By analyzing the correlation of PVR mRNA expression and the clinical characteristics of HCC patients based on the TCGA database, we found that high PVR expression was associated with a more advanced T stage (*p* = 0.031) and pathologic stage (*p* = 0.043) (Table 1).

### 3.4. Correlation and PVR-Related Gene Enrichment Analysis

**Figure 4:**
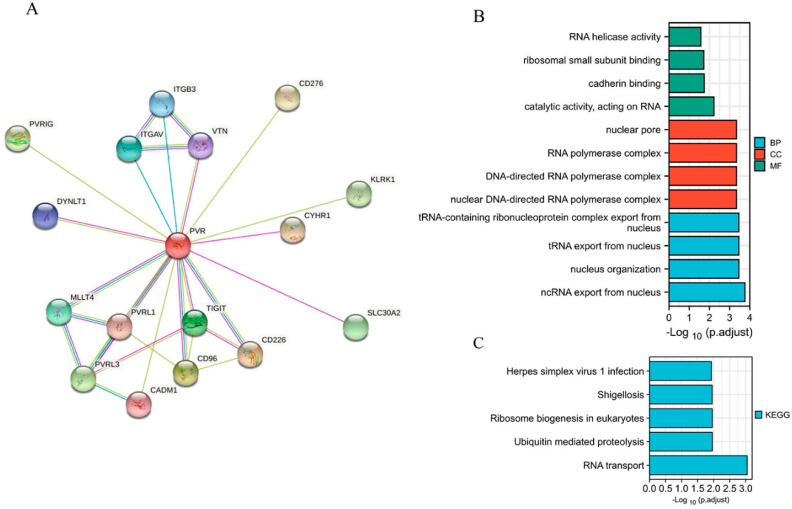
Function and pathway enrichment analysis of PVR in HCC. (**A**) PP1-binding proteins obtained by the STRING tool. (**B**) GO analysis (including BP, MF, and CC) of the top 100 genes. (**C**) KEGG pathway of the top 100 genes correlated with PVR.

When establishing a STRING network, we only considered the physical subnetwork and collected no more than 50 proteins sourced from text mining, experiences, and databases (Figure 4A). The top 100 most positively correlated genes with PVR were obtained from TCGA database. The result of GO analysis revealed that most of the genes were correlated with RNA transport, cadherin binding, and nucleus organization (Figure 4B). The KEGG data suggest that the RNA transport and ubiquitin mediated proteolysis may be related to the carcinogenic mechanism of PVR (Figure 4C).

### 3.5. Relationship between PVR Expression and Immune Scores

**Figure 5:**
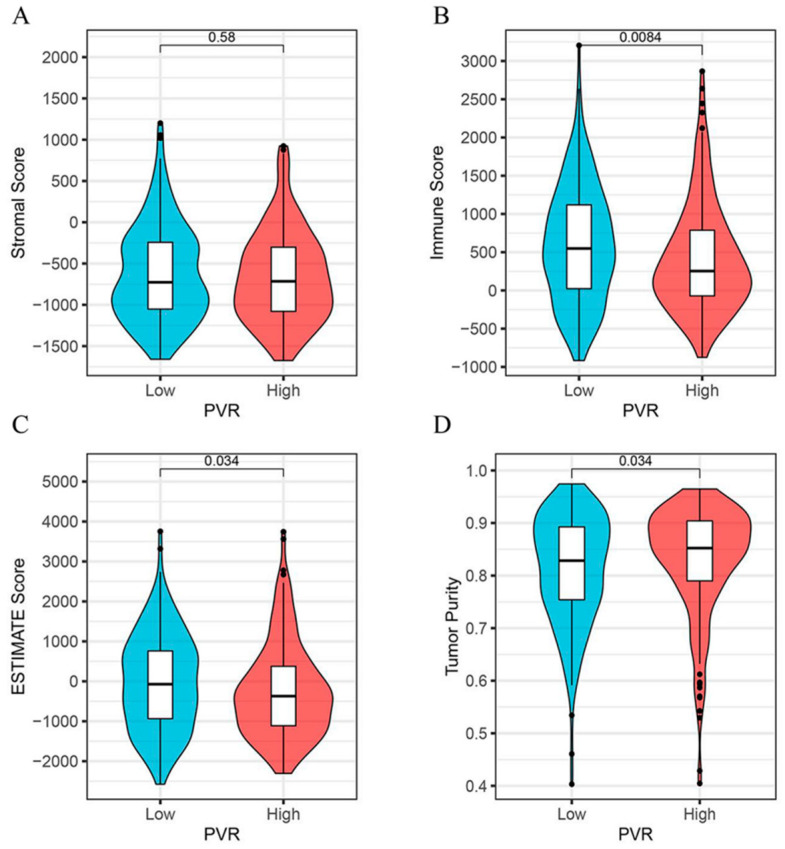
Correlation analysis between the PVR and immune scores. (**A**) The correlation between high and low PVR expression and stromal score. (**B**) The correlation between high and low PVR expression and immune score. (**C**) The correlation between high and low PVR expression and estimate score. (**D**) The correlation between high and low PVR expression and tumor purity.

As shown in Figure 5, we found that the immune score (Figure 5B) and the ESTIMATE score (Figure 5C) were both lower in the PVR high group than in the low group (*p* = 0.008, *p* = 0.034, respectively). However, the tumor purity was higher in the PVR high group when compared with the PVR low group (Figure 5D). No significant difference in the stromal score was found between the two groups (Figure 5A).

### 3.6. Relationship between PVR Expression and Immune Cell Infiltration

**Figure 6:**
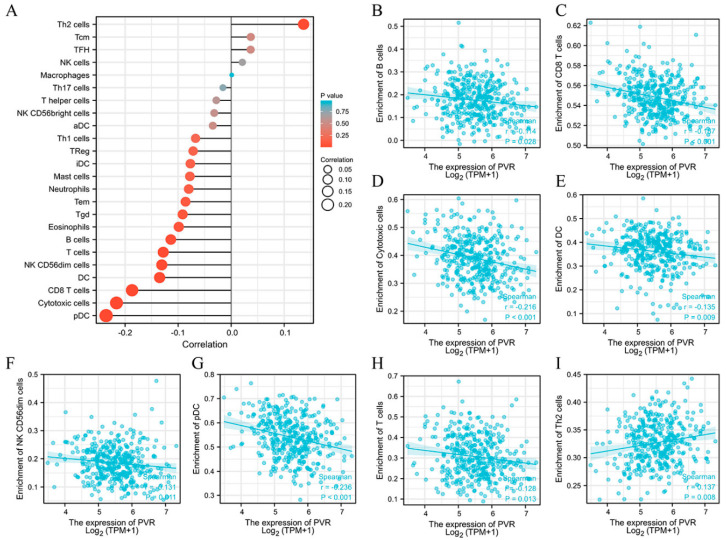
Correlation between PVR expression and immune cell infiltration. (**A**) Lollipop chart of PVR expression level in 24 immune cells. (**B**–**I**) Correlation between PVR expression and B cells, CD8+ T cells, cytotoxic cells, DC, NK CD56dim cells, pDC, T cells, and Th2 cells.

The potential association between the PVR and 24 types of immune cells were explored. There were considerable relationships between PVR expression and B cells, CD8+ T cells, cytotoxic cells, DC, NK CD56dim cells, pDC, T cells, and Th2 cells (Figure 6A). Additional studies showed that PVR expression was negatively correlated with infiltration levels of B cells (Figure 6B) (*r* = −0.114, *p* = 0.028), CD8+ T cells (Figure 6C) (*r* = −0.187, *p* < 0.001), cytotoxic cells (Figure 6D) (*r* = −0.216, *p* < 0.001), DC (Figure 6E) (*r* = −0.135, *p* = 0.009), NK CD56dim cells (Figure 6F) (*r* = −0.131, *p* = 0.011), pDC (Figure 6G) (*r* = −0.236, *p* < 0.001), and T cells (Figure 6H) (*r* = −0.128, *p* = 0.013). However, PVR expression was positively correlated with Th2 cells (Figure 6I) (*r* = 0.137, *p* = 0.008).

**Figure 7:**
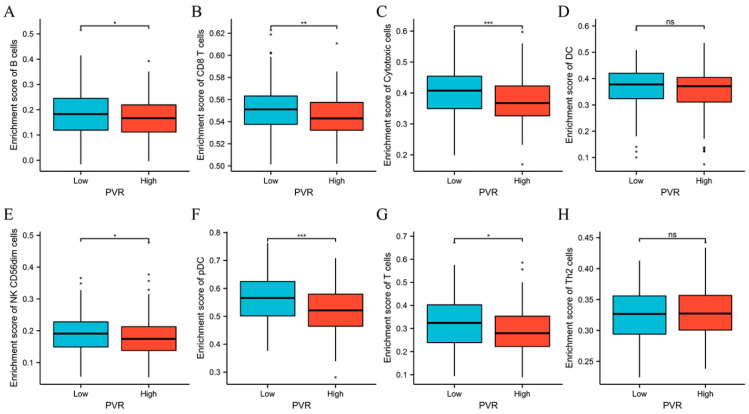
Comparison of immune cells between high and low PVR expression groups. (**A**–**H**) Histogram showing the difference of B cells, CD8+ T cells, cytotoxic cells, DC, NK CD56dim cells, pDC, T cells, and Th2 cell infiltration level between high and low PVR expression groups. * p value < 0.05; ** p value < 0.01; *** p value < 0.001.

The above results encouraged us to explore the immune infiltration differences between high and low PVR groups. We found that there were significant differences between high and low PVR groups in the infiltration of B cells, CD8+ T cells, cytotoxic cells, NK CD56dim cells, pDC, and T cells (*p* < 0.05). However, we did not observe a statistically significant difference in DC and Th2 cell infiltration between the high and low PVR groups (Figure 7).

### 3.7. Correlation between PVR Expression Levels and Immune Markers

The TIMER2.0 database was utilized to explore the relationship between PVR mRNA expression and markers of immune cells in HCC tissues. Considering that the analysis of immune infiltration was affected by tumor purity in thee clinical samples, we adjusted the analysis using purity. Table 2 shows the positive correlation between PVR expression and various immune cells, however, the majority were immunosuppressive cells.

## 4. Discussion

The above results suggest that PVR expression was significantly upregulated in a variety of cancers including HCC. However, PVR expression varied across cancers, reflecting differences in the collection methods for different data and hidden genetic mechanisms. Additionally, we found that the PVR protein was upregulated in HCC tissues than that in paracancerous tissues. In HCC, a higher PVR level was associated with poorer OS and RFS. PVR was associated with an advanced tumor stage, and more appeared in T3 + T4 stage tissues and pathologic stage III + IV tissues. Moreover, the PVR level in HCC correlated with the level of tumor-infiltrating immune cells and multiple markers of immune cells. Thus, PVR can be reasonably considered as a potential prognostic marker for HCC.

The overexpression of PVR has been reported in many cancers. PVR is a member of the cell adhesion molecules (CAMs), and its abnormal expression has an important impact on the biological behavior of tumor cells. First, PVR can promote the proliferation of tumor cells. In pancreatic cancer cells, the silencing of PVR inhibited cell proliferation, and induced cell-cycle arrest at the G2/M phase [22]. In cervical cancer, PVR expression gradually increased with the degree of cervical lesions. In vitro and in vivo, the silencing of PVR inhibited cervical cancer cell proliferation, cell viability, and induced cell-cycle arrest [23]. PVR was also upregulated in colorectal cancer. PVR knockdown inhibited colon cancer cell migration and invasion by reducing FAK, Src, and MMP−2. Additionally, PVR knockdown increased the ratio of Bax to Bcl−2, resulting in an increased proportion of apoptosis [24]. In HCC, PVR enhanced cell proliferation, migration, and invasion and promoted tumor survival and metastasis. More specifically, PVR interacted with the Src homology−2 domain and promoted Src activation, further inhibiting the p38 MAPK signaling pathway in HCC [25]. The function of PVR in the angiogenesis process for malignant tumors has gradually attracted much attention. Kinugasa M et al. [19] reported that PVR could regulate the interaction between angiogenesis factor receptor 2 (VEGFR2) and integrin αvβ3, activating the VEGFR2-mediated Rap1-Akt signaling pathway and promoting angiogenesis in tumors. In this study, we found that PVR was overexpressed in HCC tissues and negatively correlated with prognosis, supporting the potential of PVR as a biomarker to assess cancer progression and prognosis.

Tumor cells can evade immune surveillance and impair T cell function in the tumor microenvironment by expressing multiple ligands that tightly bind to inhibitory T cell receptors [26,27]. Studies have shown that the number and function of tumor-infiltrating CD4+, CD8+, or CD45+ T cells positively influence the prognosis of human cancers [28]. Therefore, enhancing the infiltration and function of tumor infiltrating lymphocyte (TILs) is a promising antitumor therapy.

As well as PD−1, LAG3, and CTLA4, increasing attention has been paid to TIGIT in recent years. TIGIT is a co-inhibitory receptor and is mainly expressed on T cells and NK cells. It is composed of an extracellular IgV domain, a type I transmembrane region, and a cytoplasmic tail containing an ITIM (immunoreceptor tyrosine-based inhibitory) motif, and an immunoglobulin tail tyrosine (ITT)-like motif [29]. TIGIT is significantly upregulated on chronically stimulated tumor-infiltrating NK and T cells, representing a hallmark of exhausted cytotoxic immune cells [30,31]. PVR on the tumor cell surface interacts with TIGIT and participates in the formation of an immunosuppressive tumor microenvironment by interacting with co-inhibitory receptors. When PVR binds to TIGIT on the surface of T cells or NK cells, it initiates the ITIM sequence in the intracellular segment. On one hand, SHP1 is recruited to block the binding of CD226 and PVR. On the other hand, ITIM directly inhibits T cell activity by blocking the MAPK pathway. TIGIT can also bind to PVR on DCs and indirectly inhibit T cell activation by modulating DC activity. More importantly, the expression of PVR/TIGIT appears to be essential for the suppressive activity of Tregs in the tumor microenvironment. In a previous study, CD8 + T cell function was found to be improved after blocking PVR/TIGIT using immunotherapies. Indeed, the blockade of PVR/TIGIT with monoclonal antibodies prevented T cell exhaustion and promoted NK cell function in mice [32]. A previous study also showed that elevated PVR/TIGIT expression was associated with a reduction in IFN-γ, but was not necessarily correlated with reduced CD8+ T cell infiltration in the tumor before immunotherapy [33]. In melanoma, NK cells with low TIGIT expression exhibited higher cytokine secretion capacity and cytotoxicity than those with high TIGIT expression [34]. In addition, myeloid-derived suppressor cell-induced NK cell inhibition was associated with the high expression of TIGIT and PVR/TIGIT interaction [35]. In this study, both the immune score and ESTIMATE score were lower in the PVR high group, suggesting a decrease in the total immune cell infiltration. Here, we also found that PVR was negatively correlated with immune cell infiltration including B cells, CD8+ T cells, cytotoxic cells, DCs, CD56dim NK cells, and pDCs. Positive correlation was observed between PVR and Th2 cell infiltration. Th2 cells and their cytokines IL−4 and IL−10 can inhibit the production of Th1 cells and their cytokines, thereby inhibiting the antitumor effect. Therefore, the high PVR expression might indicate a severe exhausted immune microenvironment in HCC.

Anti-PD1 and anti-CTLA4 therapies can induce antitumor immune responses and alter the outcome of HCC. However, resistance to these immune checkpoint inhibitors is common in clinic. Therefore, it is urgent to find new immune checkpoints. PVR and related pathway members are highlighted as key regulators of T cell function and response to cancer immunotherapy [36]. High PVR expression was found to be positively associated with an increased PD−1 + CD8+ T cells proportion within the tumor, which was also associated with early progression in melanoma patients treated with anti-PD1. Furthermore, researchers have demonstrated that the silencing of PVR in melanoma increased sensitivity to combined anti-PD1/CTLA4 therapy, suggesting that targeting PVR could serve as a complement to current immunosuppressive therapies [33]. Consistent with previous studies, positive correlations between PVR and exhausted T-cell markers such as TIM3 and PD1 were observed in this study. Therefore, targeting PVR immunotherapy appears to be a promising option for the treatment of HCC. However, few studies have been conducted on the effect of the PVR antibody either in vitro or in vivo. Therefore, the role of PVR in HCC immunotherapy needs to be further studied.

## 5. Conclusions

Our findings suggest that PVR might be a potential prognostic marker for HCC and can be used to determine the infiltration of immune cells in cancer tissues. The relatively higher level of PVR in HCC suggests a shorter survival time and a higher risk of tumor recurrence. Therefore, close medical monitoring is critical for these patients. However, further long-term follow-up and a larger patient-cohort validation studies are needed to definitively validate PVR as a predictor.

## Figures and Tables

**Table 1:** Correlation of PVR mRNA expression and the clinical characteristics of HCC patients.

Characteristic	Low expression of PVR	High Expression of PVR	*p* Value
*n*	187	187	
Gender, *n* (%)			0.825
Female	59 (15.8%)	62 (16.6%)	
Male	128 (34.2%)	125 (33.4%)	
Age, *n* (%)			0.961
≤60	89 (23.9%)	88 (23.6%)	
>60	97 (26.0%)	99 (26.5%)	
Race, *n* (%)			0.760
Asian	76 (21.0%)	84 (23.2%)	
Black or African American	8 (2.2%)	9 (2.5%)	
White	95 (26.2%)	90 (24.9%)	
T stage, *n* (%)			0.031
T1 + T2	146 (39.5%)	131(35.4%)	
T3 + T4	37 (10.0%)	56 (15.1%)	
N stage, *n* (%)			0.628
N0	117 (45.3%)	137 (53.1%)	
N1	1 (0.4%)	3 (1.2%)	
M stage, *n* (%)			0.337
M0	122 (44.9%)	146 (53.7%)	
M1	3 (1.1%)	1 (0.4%)	
Pathologic stage, *n* (%)			0.043
Stage I + Stage II	139 (39.7%)	121 (34.6%)	
Stage III + Stage IV	37 (10.6%)	53 (15.1%)	
Histologic grade, *n* (%)			0.778
G1	30 (8.1%)	25 (6.8%)	
G2	90 (24.4%)	88 (23.8%)	
G3	59 (16%)	65 (17.6%)	
G4	5 (1.4%)	7 (1.9%)	
Child–Pugh grade, *n* (%)			0.910
A	108 (44.8%)	111 (46.1%)	
B	10 (4.1%)	11 (4.6%)	
C	1 (0.4%)	0 (0%)	
Vascular invasion, *n* (%)			0.561
No	103 (32.4%)	105 (33%)	
Yes	59 (18.6%)	51 (16%)	

**Table 2:** Correlation analysis between PVR and related markers of immune cells in TIMER2.0.

Description	Gene Markers	None		Purity	
		Core	*p*	Core	*p*
CD8+ T cell	CD8A	−0.045	0.389	0.056	0.300
	CD8B	−0.088	0.091	−0.001	0.991
T cell (general)	CD3D	−0.071	0.172	0.001	0.982
	CD3E	−0.073	0.162	0.035	0.521
	CD2	−0.064	0.220	0.037	0.494
B cell	CD19	−0.055	0.292	0.015	0.784
	CD79A	−0.064	0.217	0.026	0.630
Monocyte	CD86	0.022	0.670	0.136	*
	CSF1R	−0.007	0.891	0.112	*
TAM	CCL2	0.003	0.960	0.098	0.068
	CD68	0.027	0.600	0.103	0.057
	IL10	0.005	0.929	0.102	0.058
M1 Macrophage	iNOS	0.143	**	0.161	**
	IRF5	0.228	***	0.232	***
	PTGS2	0.115	*	0.234	***
M2 Macrophage	CD163	0.045	0.388	0.171	**
	VSIG4	0.092	*	0.214	***
	MS4A4A	0.002	0.969	0.125	*
Natural killer cell	KIR2DL1	−0.043	0.406	−0.036	0.505
	KIR2DL3	−0.051	0.331	−0.019	0.722
	KIR2DL4	−0.034	0.517	0.017	0.756
	KIR3DL1	−0.037	0.475	−0.011	0.844
	KIR3DL2	−0.056	0.286	−0.011	0.840
	KIR3DL3	−0.087	0.094	−0.066	0.224
	KIR2DS4	−0.021	0.687	−0.006	0.912
Dendritic cell	HLA-DPB1	−0.082	0.114	0.010	0.859
	HLA-DQB1	−0.071	0.170	0.011	0.837
	HLA-DRA	−0.055	0.292	0.045	0.400
	HLA-DPA1	−0.053	0.307	0.054	0.317
	BDCA−1(CD1C)	−0.010	0.844	0.053	0.328
	BDCA−4(NRP1)	0.143	**	0.177	**
	CD11c(ITGAX)	0.116	*	0.237	***
Th1	TBX21	−0.088	0.089	0.009	0.866
	STAT4	0.097	0.061	0.145	**
	STAT1	0.165	**	0.224	***
	TNF	0.040	0.442	0.165	*
	INF-α(IL28B)	−0.037	0.472	0.061	0.260
Th2	GATA3	−0.006	0.905	0.110	*
	STAT6	0.160	**	0.183	***
	STAT5A	0.135	**	0.206	***
	IL13	0.088	0.092	0.119	*
Tfh	BCL6	0.147	**	0.158	**
	IL21	−0.065	0.210	−0.038	0.483
Th17	STAT3	0.234	***	0.307	***
	IL17A	−0.021	0.689	−0.025	0.649
Treg	FOXP3	0.116	*	0.149	***
	CCR8	0.149	**	0.238	***
	STAT5B	0.325	***	0.332	***
	TGFB1	0.058	0.264	0.122	*
T cell exhaustion	PD−1	−0.032	0.542	0.146	**
	CTLA4	−0.020	0.700	0.058	0.281
	LAG3	−0.030	0.568	0.014	0.798
	TIM3	0.042	0.417	0.162	**
	GZMB	−0.119	*	−0.046	0.394
Neutrophils	CD66b (CEACAM8)	0.089	0.088	0.107	*
	CD11b (ITGAM)	0.065	0.213	0.134	*
	CCR7	−0.065	0.214	0.052	0.338

* *p* < 0.05, ** *p* < 0.01, *** *p* < 0.001.

## Data Availability

All data are available on request.

## Supplementary Materials

The following supporting information can be downloaded at: https://www.mdpi.com/article/10.3390/diagnostics12122953/s1, Figure S1. PVR has prognostic value in other malignant tumors. (A) OS of esophageal squamous cell carcinoma between the high PVR group and low PVR group. (B) OS of lung adenocarcinoma between the high and low PVR group.

## References

[1] Sung H., Ferlay J., Siegel R.L., Laversanne M., Soerjomataram I., Jemal A., Bray F. (2021). Global Cancer Statistics 2020: GLOBOCAN Estimates of Incidence and Mortality Worldwide for 36 Cancers in 185 Countries. CA Cancer J. Clin..

[2] Ferlay J., Colombet M., Soerjomataram I., Dyba T., Randi G., Bettio M., Gavin A., Visser O., Bray F. (2018). Cancer incidence and mortality patterns in Europe: Estimates for 40 countries and 25 major cancers in 2018. Eur. J. Cancer.

[3] Long J., Wang A., Bai Y., Lin J., Yang X., Wang D., Yang X., Jiang Y., Zhao H. (2019). Development and validation of a TP53-associated immune prognostic model for hepatocellular carcinoma. EBioMedicine.

[4] Fujiwara N., Friedman S.L., Goossens N., Hoshida Y. (2018). Risk factors and prevention of hepatocellular carcinoma in the era of precision medicine. J. Hepatol..

[5] Huang D.Q., El-Serag H.B., Loomba R. (2021). Global epidemiology of NAFLD-related HCC: Trends, predictions, risk factors and prevention. Nat. Rev. Gastroenterol. Hepatol..

[6] Li W., Liu K., Chen Y., Zhu M., Li M. (2021). Role of Alpha-fetoprotein in hepatocellular carcinoma drug resistance. Curr. Med. Chem..

[7] Pardoll D.M. (2012). The blockade of immune checkpoints in cancer immunotherapy. Nat. Rev. Cancer.

[8] Topalian S.L., Drake C.G., Pardoll D.M. (2015). Immune checkpoint blockade: A common denominator approach to cancer therapy. Cancer Cell.

[9] Chaudhary B., Elkord E. (2016). Regulatory T Cells in the tumor microenvironment and cancer progression: Role and therapeutic targeting. Vaccines.

[10] Sasidharan N.V., Elkord E. (2018). Immune checkpoint inhibitors in cancer therapy: A focus on T-regulatory cells. Immunol. Cell. Biol..

[11] Kakunaga S., Ikeda W., Shingai T., Fujito T., Yamada A., Minami Y., Imai T., Takai Y. (2004). Enhancement of serum- and platelet-derived growth factor-induced cell proliferation by Necl-5/Tage4/poliovirus receptor/CD155 through the Ras-Raf-MEK-ERK signaling. J. Biol. Chem..

[12] Oda T., Ohka S., Nomoto A. (2004). Ligand stimulation of CD155alpha inhibits cell adhesion and enhances cell migration in fibroblasts. Biochem. Biophys. Res. Commun..

[13] Lange R., Peng X., Wimmer E., Lipp M., Bernhardt G. (2001). The poliovirus receptor CD155 mediates cell-to-matrix contacts by specifically binding to vitronectin. Virology.

[14] Reymond N., Imbert A.-M., Devilard E., Fabre S., Chabannon C., Xerri L., Farnarier C., Cantoni C., Bottino C., Moretta A. (2004). DNAM-1 and PVR Regulate Monocyte Migration through Endothelial Junctions. J. Exp. Med..

[15] Braun M., Aguilera A.R., Sundarrajan A., Corvino D., Stannard K., Krumeich S., Das I., Lima L.G., Guzman L.G.M., Li K. (2020). CD155 on Tumor Cells Drives Resistance to Immunotherapy by Inducing the Degradation of the Activating Receptor CD226 in CD8+ T Cells. Immunity.

[16] Liu L., You X., Han S., Sun Y., Zhang J., Zhang Y. (2021). CD155/TIGIT, a novel immune checkpoint in human cancers (Review). Oncol. Rep..

[17] Molfetta R., Zitti B., Lecce M., Milito N.D., Stabile H., Fionda C., Cippitelli M., Gismondi A., Santoni A., Paolini R. (2020). CD155: A multi-functional molecule in tumor progression. Int. J. Mol. Sci..

[18] Nakai R., Maniwa Y., Tanaka Y., Nishio W., Yoshimura M., Okita Y., Ohbayashi C., Satoh N., Ogita H., Takai Y. (2010). Overexpression of Necl-5 correlates with unfavorable prognosis in patients with lung adenocarcinoma. Cancer Sci..

[19] Kinugasa M., Amano H., Satomi-Kobayashi S., Nakayama K., Miyata M., Kubo Y., Nagamatsu Y., Kurogane Y., Kureha F., Yamana S. (2012). Necl-5/poliovirus receptor interacts with VEGFR2 and regulates VEGF-induced angiogenesis. Circ. Res..

[20] Chauvin J.M., Zarour H.M. (2020). TIGIT in cancer immunotherapy. J. Immunother. Cancer.

[21] Zhao K., Ma Z., Zhang W. (2022). Comprehensive Analysis to Identify SPP1 as a Prognostic Biomarker in Cervical Cancer. Front. Genet..

[22] Nishiwada S., Sho M., Yasuda S., Shimada K., Yamato I., Akahori T., Kinoshita S., Nagai M., Konishi N., Nakajima Y. (2015). Clinical significance of CD155 expression in human pancreatic cancer. Anticancer Res..

[23] Liu L., Wang Y., Geng C., Wang A., Han S., You X., Sun Y., Zhang J., Lu W., Zhang Y. (2021). CD155 promotes the progression of cervical cancer cells through AKT/mTOR and NF-κB pathways. Front. Oncol..

[24] Zheng Q., Wang B., Gao J., Xin N., Wang W., Song X., Shao Y., Zhao C. (2018). CD155 knockdown promotes apoptosis via AKT/Bcl-2/Bax in colon cancer cells. J. Cell. Mol. Med..

[25] Jin A.L., Zhang C.Y., Zheng W.J., Xian J.R., Yang W.J., Liu T., Chen W., Li T., Wang B.L., Pan B.S. (2022). CD155/SRC complex promotes hepatocellular carcinoma progression via inhibiting the p38 MAPK signalling pathway and correlates with poor prognosis. Clin. Transl. Med..

[26] Iwai Y., Ishida M., Tanaka Y., Okazaki T., Honjo T., Minato N. (2002). Involvement of PD-L1 on tumor cells in the escape from host immune system and tumor immunotherapy by PD-L1 blockade. Proc. Natl. Acad. Sci. USA.

[27] Dong H., Strome S.E., Salomao D.R., Tamura H., Hirano F., Flies D.B., Roche P.C., Lu J., Zhu G., Tamada K. (2002). Tumor-associated B7-H1 promotes T-cell apoptosis: A potential mechanism of immune evasion. Nat. Med..

[28] Huang X., Zhang G., Tang T., Liang T. (2021). Identification of tumor antigens and immune subtypes of pancreatic adenocarcinoma for mRNA vaccine development. Mol. Cancer.

[29] Mahnke K., Enk A.H. (2016). TIGIT-CD155 Interactions in melanoma: A novel co-inhibitory pathway with potential for clinical intervention. J. Investig. Dermatol..

[30] Johnston R.J., Comps-Agrar L., Hackney J., Yu X., Huseni M., Yang Y., Park S., Javinal V., Chiu H., Irving B. (2014). The immunoreceptor TIGIT regulates antitumor and antiviral CD8(+) T cell effector function. Cancer Cell.

[31] Zhang Q., Bi J., Zheng X., Chen Y., Wang H., Wu W., Wang Z., Wu Q., Peng H., Wei H. (2018). Blockade of the checkpoint receptor TIGIT prevents NK cell exhaustion and elicits potent anti-tumor immunity. Nat. Immunol..

[32] Blake S.J., Dougall W.C., Miles J.J., Teng M.W., Smyth M.J. (2016). Molecular pathways: Targeting CD96 and TIGIT for cancer immunotherapy. Clin. Cancer Res..

[33] Lepletier A., Madore J., O’Donnell J.S., Johnston R.L., Li X.-Y., McDonald E., Ahern E., Kuchel A., Eastgate M., Pearson S.-A. (2020). Tumor CD155 expression is associated with resistance to anti-PD1 immunotherapy in metastatic melanoma. Clin. Cancer. Res..

[34] Chauvin J.-M., Ka M., Pagliano O., Menna C., Ding Q., DeBlasio R., Sanders C., Hou J., Li X.-Y., Ferrone S. (2020). IL15 Stimulation with TIGIT blockade reverses CD155-mediated NK-Cell dysfunction in melanoma. Clin. Cancer. Res..

[35] Sarhan D., Cichocki F., Zhang B., Yingst A., Spellman S.R., Cooley S., Verneris M.R., Blazar B.R., Miller J.S. (2016). Adaptive NK cells with low TIGIT expression are inherently resistant to myeloid-derived suppressor cells. Cancer Res..

[36] Chiu D.K.-C., Yuen V.W.-H., Cheu J.W.-S., Wei L.L., Ting V., Fehlings M., Sumatoh H., Nardin A., Newell E.W., Ng I.O.-L. (2020). Hepatocellular Carcinoma Cells Up-regulate PVRL1, Stabilizing PVR and Inhibiting the Cytotoxic T-Cell Response via TIGIT to Mediate Tumor Resistance to PD1 Inhibitors in Mice. Gastroenterology.

