# Supplementary figures and images for: PVR—A Prognostic Biomarker Correlated with Immune Cell Infiltration in Hepatocellular Carcinoma
Source: Diagnostics (Basel). 2022 Nov 25;12(12):2953. doi: 10.3390/diagnostics12122953 (PMC9777148; doi:10.3390/diagnostics12122953)

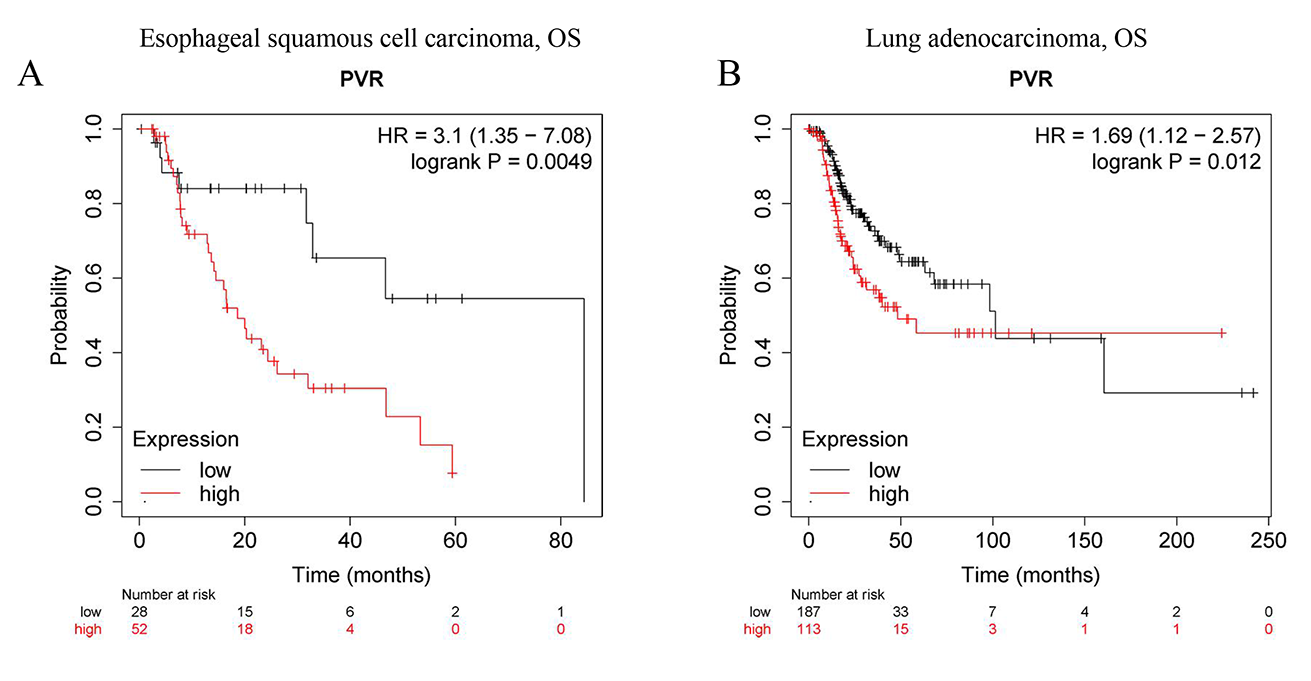

Supplement: Supplementary file 1 [file diagnostics-12-02953-s001.zip › diagnostics-1991194-Supplementary.tif]
